# Artificial intelligence in imaging for liver disease diagnosis

**DOI:** 10.3389/fmed.2025.1591523

**Published:** 2025-04-25

**Authors:** Chenglong Yin, Huafeng Zhang, Jin Du, Yingling Zhu, Hua Zhu, Hongqin Yue

**Affiliations:** ^1^Department of Gastroenterology, Affiliated Hospital 6 of Nantong University, Yancheng Third People's Hospital, Yancheng, Jiangsu, China; ^2^Affiliated Yancheng Hospital, School of Medicine, Southeast University, Yancheng, Jiangsu, China; ^3^Yancheng Ruikang Hospital, Yancheng, Jiangsu, China; ^4^Department of Science and Education, Affiliated Hospital 6 of Nantong University, Yancheng Third People's Hospital, Yancheng, Jiangsu, China

**Keywords:** liver diseases, artificial intelligence, imaging, diagnosis, clinical applications

## Abstract

Liver diseases, including hepatitis, non-alcoholic fatty liver disease (NAFLD), cirrhosis, and hepatocellular carcinoma (HCC), remain a major global health concern, with early and accurate diagnosis being essential for effective management. Imaging modalities such as ultrasound (US), computed tomography (CT), and magnetic resonance imaging (MRI) play a crucial role in non-invasive diagnosis, but their sensitivity and diagnostic accuracy can be limited. Recent advancements in artificial intelligence (AI) have improved imaging-based liver disease assessment by enhancing pattern recognition, automating fibrosis and steatosis quantification, and aiding in HCC detection. AI-driven imaging techniques have shown promise in fibrosis staging through US, CT, MRI, and elastography, reducing the reliance on invasive liver biopsy. For liver steatosis, AI-assisted imaging methods have improved sensitivity and grading consistency, while in HCC detection and characterization, AI models have enhanced lesion identification, classification, and risk stratification across imaging modalities. The growing integration of AI into liver imaging is reshaping diagnostic workflows and has the potential to improve accuracy, efficiency, and clinical decision-making. This review provides an overview of AI applications in liver imaging, focusing on their clinical utility and implications for the future of liver disease diagnosis.

## 1 Introduction

Liver diseases, such as hepatitis, non-alcoholic fatty liver disease (NAFLD), cirrhosis, and hepatocellular carcinoma (HCC), continue to pose significant health challenges worldwide (1–3). In 2022, viral hepatitis alone accounts for approximately 1.3 million deaths, making it one of the leading causes of infectious mortality (4–6). NAFLD, affecting nearly a quarter of the global population, is becoming more prevalent due to increasing rates of obesity and metabolic syndrome (7, 8). As these conditions advance, the burden of cirrhosis and HCC continues to rise (9–11), exacerbating healthcare challenges.

**Figure 1:**
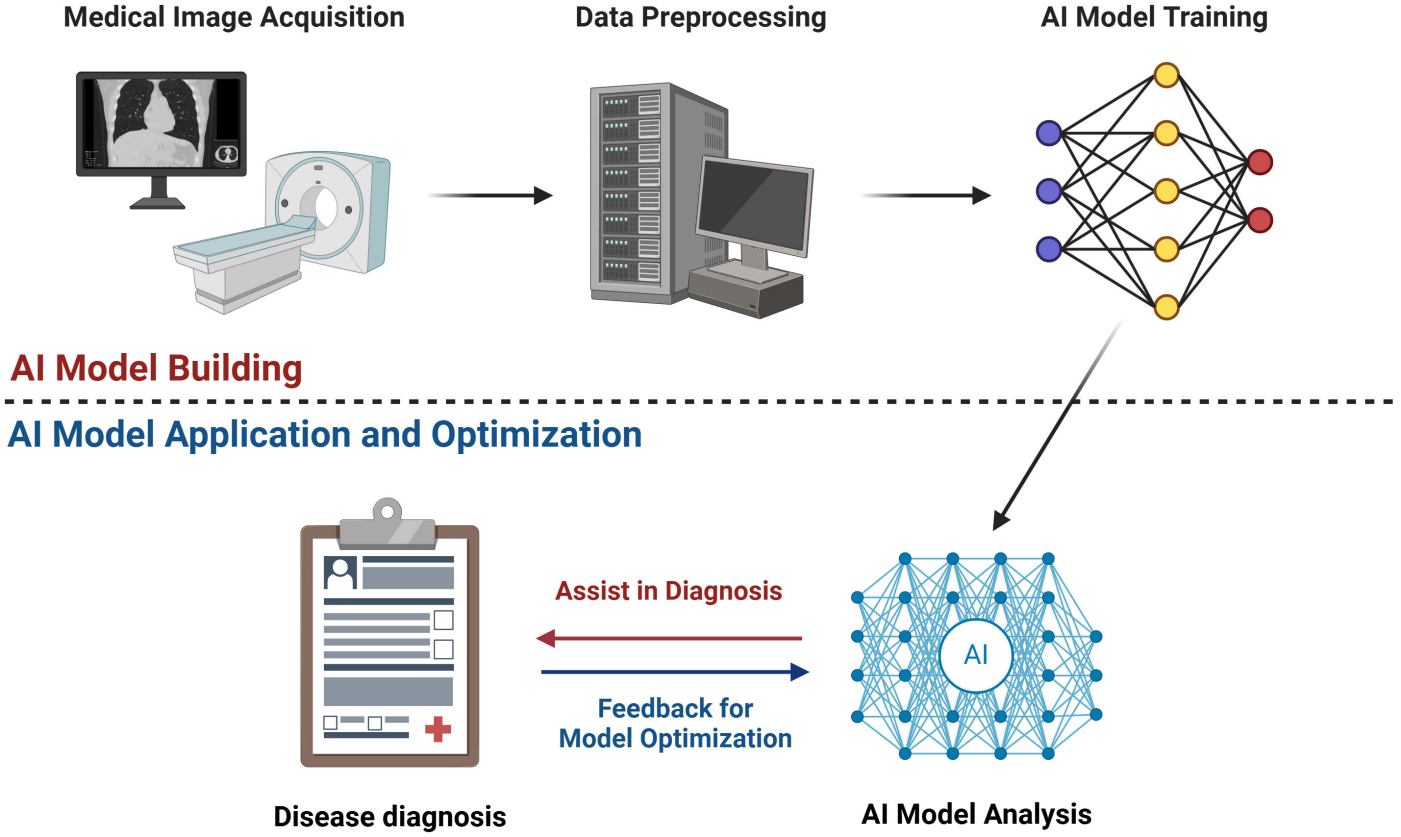
AI model building, application, and optimization.

The early and precise diagnosis of hepatic disorders is crucial for optimizing clinical outcomes and therapeutic strategies (12). Imaging modalities such as ultrasound (US), computed tomography (CT), and magnetic resonance imaging (MRI) provide non-invasive insights into liver pathology (13–16). However, conventional imaging approaches face inherent limitations, including interobserver variability, reduced sensitivity in early disease stages, and subjective interpretation of subtle pathological changes (17, 18). Artificial intelligence (AI) is transforming medical imaging (19, 20) by enabling automated image analysis, improving pattern recognition, and enhancing predictive modeling (21–23). AI-driven imaging techniques have demonstrated significant potential in improving the detection, classification, and quantification of liver fibrosis, steatosis, and HCC (24–28). Through the use of deep learning and radiomics, AI enhances diagnostic accuracy and efficiency (29, 30), aiding clinicians in making more informed treatment decisions (Figure 1).

Given the critical role of imaging in liver disease diagnosis, the integration of AI into radiology represents a paradigm shift in diagnostic methodologies. This review explores the integration of AI into liver imaging, emphasizing its applications in fibrosis assessment, steatosis quantification, and HCC detection. Additionally, we discuss the clinical implications of AI-assisted imaging and the challenges associated with its adoption in routine clinical practice.

## 2 Liver fibrosis and cirrhosis

Liver fibrosis, and its advanced stage cirrhosis, are progressive conditions with significant clinical implications, including an increased risk of portal hypertension and HCC. Imaging plays a pivotal role in non-invasive fibrosis staging, reducing reliance on liver biopsy, which is invasive and prone to sampling error (31). AI-driven imaging techniques utilizing US, CT, MRI, and elastography have demonstrated notable promise in improving diagnostic accuracy and fibrosis staging (Table 1).

**Table 1:** The performance of AI in imaging diagnosis of liver fibrosis and cirrhosis.

**Imaging technology**	**Artificial intelligence approaches**	**Role**	**Diagnostic performance**	**References**
B-mode US	DCNN	Fibrosis staging	High diagnostic accuracy	(32)
US	NN	Fibrosis diagnosis	AUC = 0.92	(33)
CT	DL	Fibrosis staging	Overall accuracy 79.4%; AUC: F4 = 0.95, ≥F3 = 0.97, ≥F2 = 0.96	(34)
CT	DL	Fibrosis quantification	AUC: F4 = 0.73, ≥F3 = 0.76, ≥F2 = 0.74	(35)
MRI	CNN	Cirrhosis detection	High accuracy for portal hypertension	(36)
MRI	DCNN	Fibrosis staging	AUC: F4 = 0.84, ≥F3 = 0.84, ≥F2 = 0.85	(37)
Elastography	SWE	≥F2 fibrosis detection	AUC = 0.89 vs. 0.74	(38)
Elastography	DL	Fibrosis staging	Significantly better than conventional liver fibrosis index method	(39)
Elastography	DL	Fibrosis staging	AUC: F4 = 0.97, ≥F3 = 0.98, ≥F2 = 0.85	(40)

### 2.1 Ultrasound-based methods

The integration of AI with ultrasonography has demonstrated significant potential for non-invasive fibrosis staging. Lee et al. developed a deep convolutional neural network (DCNN) based on US data from 3,446 patients to stage liver fibrosis, and the high diagnostic accuracy was confirmed through internal and external test sets with 266 and 572 patients, respectively (32). In another study, five US variables were used as input for a neural network, including liver parenchyma, spleen thickness, hepatic vein waveform, hepatic artery pulsatilite index, and damping index (33). The network diagnosed liver fibrosis, achieving an area under the curve (AUC) of 0.92.

### 2.2 Computed tomography-based methods

CT imaging provides objective fibrosis assessment, and AI-driven models have further refined its diagnostic potential. Choi et al. used a deep learning system (DLS) to analyze contrast-enhanced CT images of 7,461 patients with histologically confirmed liver fibrosis stages. The DLS showed an overall diagnostic accuracy of 79.4%, with AUCs of 0.95, 0.97, and 0.96 for cirrhosis (F4), advanced fibrosis (≥F3), and significant fibrosis (≥F2) stages, respectively (34). Another study applied a deep learning model based on magnified CT images. The fibrosis scores obtained from deep learning based on CT images (F_DLCT_ scores) showed significant correlation with the pathological staging of liver fibrosis. Using F_DLCT_ scores, the AUCs for predicting stages F4, ≥F3, and ≥F2 were 0.73, 0.76, and 0.74, respectively (35).

### 2.3 Magnetic resonance imaging-based methods

MRI-based AI models have advanced fibrosis staging by integrating radiomics and deep learning approaches. Liu et al. developed and validated a radiomics signature, radiomics hepatic venous pressure gradient (rHVPG), as a non-invasive and accurate tool for detecting cirrhosis. This tool can aid in the rapid and non-invasive identification of cirrhosis and portal hypertension and has been extended to MRI (36). In addition, Yasaka et al. conducted a retrospective study to assess the performance of a DCNN model based on gadoxetic acid–enhanced hepatobiliary phase MRI. The fibrosis score obtained through deep learning showed strong correlation with pathological fibrosis staging. The AUCs for diagnosing F4, ≥F3, and ≥F2 were 0.84, 0.84, and 0.85, respectively (37).

### 2.4 Elastography-based methods

In addition to US, CT, and MRI, elastography has clear advantages in detecting liver fibrosis and cirrhosis. The automated framework based on shear wave elastography can provide better accuracy in detecting ≥F2 fibrosis compared to conventional stiffness, with AUCs of 0.89 and 0.74, respectively (38). In another study, 11 image features were extracted from real-time tissue elastography software. Data were processed using four classic classifiers, and it was showed that this method performed significantly better than conventional liver fibrosis index method (39). Wang et al. also developed a deep learning radiomics of elastography (DLRE). As a non-invasive tool, DLRE achieved an AUC of 0.97 for F4, 0.98 for ≥F3, and 0.85 for ≥F2 (40).

## 3 Liver steatosis

Diagnosing and grading liver steatosis remain challenging due to the limitations of conventional imaging techniques. While US is widely used, its sensitivity in detecting mild steatosis is limited and highly operator-dependent. CT provides objective liver fat quantification, but exposes patients to ionizing radiation, making it suboptimal for routine screening. The integration of AI with these modalities has significantly improved liver fat quantification, enhancing diagnostic consistency and reducing reliance on invasive liver biopsy (Table 2).

**Table 2:** The performance of AI in imaging diagnosis of liver steatosis.

**Imaging technology**	**Artificial intelligence approaches**	**Role**	**Diagnostic performance**	**References**
US	ELM	Fatty liver risk stratification	40% faster than SVM	(41)
US	DCNN	NAFLD evaluation	Outperformed traditional methods	(42)
US	MEL	Fat content assessment	Superior to single-view models	(43)
US	CNN	Steatosis detection	AUC = 0.93	(44)
US	CNN + MEL	Fat fraction estimation	Comparable to MRI	(45)
US	DL	NAFLD diagnosis	96% accuracy; high correlation with MRI-PDFF	(46)
US	DL	Steatosis severity	Enhance the classification of liver steatosis	(47)
US	DL	Steatosis severity	Outperformed SVM	(48)
US	DL	Steatosis grading	High classification accuracy	(50)
US	CNN	Classify fatty liver images	AUC = 0.9999	(49)
US	DL	Steatosis grading	AUC: mild = 0.85, moderate = 0.91, severe = 0.93	(51)
US	DL	Steatosis diagnosis	99.91% diagnostic accuracy	(52)
CT	DL	Steatosis grading	93.33% diagnostic accuracy	(53)
CT	DL	NAFLD severity	Outstanding diagnostic performance	(54)
CT	DL	Automated fat quantification	Effective for asymptomatic populations	(55)
CT	NLP	Multi-modal report analysis	>90% recall/accuracy	(56)

### 3.1 Ultrasound-based methods

AI-enhanced US techniques have shown improved sensitivity in detecting and quantifying liver fat content. Kuppili et al. demonstrated the potential of the extreme learning machine model in stratifying the risk of fatty liver from liver US images, outperforming traditional methods such as support vector machines (SVM) and improving diagnostic speed by 40% (41). Similarly, Byra et al. introduced a DCNN method for evaluating NAFLD (42). In a study, a multi-view ensemble model was shown to perform more accurately than single-view models in assessing liver fat (43). Constantinescu et al. contributed to the field by applying two convolutional neural network (CNN) models for steatosis detection, with Inception-v3 achieving an impressive AUC of 0.93 (44). Kim et al. further combined the VGG19 CNN with multi-view US images, demonstrating that deep learning could replace MRI in detecting fatty liver and estimating fat fraction (45). Another study found deep learning algorithms utilizing radiofrequency US data demonstrated 96% diagnostic accuracy in diagnosing NAFLD, with fat fraction estimations highly correlated to MRI-derived proton density fat fraction (46).

The rapid advancements in AI have also elevated the diagnostic capabilities of US in determining the severity of liver steatosis. Destrempes et al. combined quantitative US with shear wave elastography and a random forest model, enhancing the classification of liver steatosis (47). Further studies have demonstrated the superiority of random forest classifiers over SVM in assessing liver steatosis severity (48). Zamanian et al. introduced a neural network-based model that achieved a remarkable AUC of 0.9999 in classifying fatty liver images (49). Furthermore, the ResNet-50 v2 model exhibited high classification accuracy for varying levels of liver steatosis (50), while Li et al. employed a deep learning algorithm to quantitatively score liver steatosis with AUCs of 0.85, 0.91, and 0.93 for mild, moderate, and severe grades, respectively (51). Rhyou et al. developed a fully automated model that achieved 99.91% diagnostic accuracy (52). A computer-aided diagnosis (CAD) system achieved a classification accuracy of 93.33% for steatosis grading (53). Cao et al. demonstrated outstanding diagnostic performance of deep learning in US for evaluating the severity of NAFLD (54).

### 3.2 Computed tomography-based methods

The application of AI in conjunction with CT holds significant promise for advancing the diagnosis of liver steatosis. Graffy et al. leveraged deep learning algorithms to automatically assess the CT values of the liver, offering an effective and non-invasive method for evaluating fatty liver in asymptomatic populations undergoing routine screening (55). Similarly, Redmond et al. applied natural language processing to develop an algorithm capable of accurately identifying fatty liver disease, achieving over 90% recall and accuracy across US, CT, and MRI reports (56). Furthermore, an automated liver attenuation regions of interest-based method has been introduced, showing excellent performance in detecting NAFLD on CT scans (57).

## 4 Hepatocellular carcinoma

The application of AI in HCC imaging has led to notable advancements in lesion detection, classification, and risk stratification. Traditional imaging methods often struggle to distinguish malignant from benign hepatic lesions, particularly in early-stage HCC or atypical presentations. AI-driven approaches have improved diagnostic precision, reduced interobserver variability, and enabled automated risk assessment across US, CT, and MRI (Table 3).

**Table 3:** The performance of AI in imaging diagnosis of hepatocellular carcinoma.

**Imaging technology**	**Artificial intelligence approaches**	**Role**	**Diagnostic performance**	**References**
US	NN	FLL classification	95% accuracy	(58)
US	DL	Disease staging	96.6% accuracy	(59)
US	DCNN	FLL classification	Mean AUC = 0.935/0.916	(60)
US	CNN	HCC vs. cirrhotic parenchyma	Outperformed ML models	(61)
US	DCNN	Benign vs. malignant lesions	AUC = 0.924	(62)
US	Multiple kernel learning	FLL classification	Classification accuracy of 90.41%	(67)
US	CEUS + SVM	Atypical HCC vs. FNH	Effective differentiation	(68)
US	CEUS	Preoperative grading	Superior AUC	(69)
CT	CNN	Lesion detection/classification	93.4% recall; 82.5% binary accuracy	(70)
CT	DL	HCC vs. other malignancies	AUC = 0.92	(71)
CT	CNN	Tumor detection	98.3% classification accuracy and 78% tumor detection rate	(72)
CT	CNN+FCN+SSD	Tumor detection	100% detection accuracy	(73)
CT	CNN + SVM	HCC vs. ICC	88% accuracy	(74)
CT	DL	Tumor detection	98.39%−100% detection accuracy	(75)
CT	CNN	Tumor segmentation	Dice = 80.06%; Precision = 82.67%	(78)
CT	FCN	Segmentation (multi-phase CT)	Volume overlap error: 15.6% → 8.1%	(79)
CT	DL	Segmentation	85% false-positive reduction; Dice = 69%	(80)
CT	DL	Differentiate HCC from other FLLs	Diagnostic accuracy with safety considerations	(83)
CT	CNN	Recurrence monitoring	Detection rate: 72% → 86%	(84)
MRI	DL	Lesion classification	77% overall accuracy	(85)
MRI	3D CNN	Lesion classification	83% accuracy	(88)
MRI	CNN	LI-RADS grading	90% accuracy; AUC = 0.95	(91)
MRI	DL	HCC differentiation	AUC = 0.999	(92)
MRI	CNN	Automated HCC delineation	Demonstrated feasibility	(93)
MRI	3D CNN	Atypical HCC classification	Overall accuracy = 87.3%; AUC = 0.912	(94)

### 4.1 Ultrasound-based methods

AI-based approaches have been developed to enhance the performance of US imaging, particularly in lesion classification, risk stratification, and preoperative assessment. Recent studies have demonstrated the effectiveness of deep learning models in focal liver lesion (FLLs) classification. A neural network ensemble-based CAD model achieved a classification accuracy of 95% (58). Similarly, Bharti et al. developed an ensemble classifier-based model that improved liver disease staging accuracy to 96.6% (59), while Schmauch et al. introduced a supervised deep learning model that achieved mean AUC values of 0.935 and 0.916, respectively (60). Notably, a CNN model using US images has outperformed conventional machine learning models in distinguishing HCC from cirrhotic parenchyma (61). Additionally, for distinguishing between benign and malignant liver lesions, a DCNN model based on US images achieved an AUC of 0.924, surpassing the diagnostic accuracy of experienced radiologists (62).

Ultrasomics, which integrates radiomic feature extraction with machine learning algorithms, has shown promise in differentiating primary and metastatic liver tumors. Logistic regression classifier leveraging ultrasomic features have demonstrated superior performance compared to conventional imaging methods (63). Furthermore, ultrasomics combined with clinical data has significantly improved the accuracy of preoperative pathological grading of HCC (64) and enhanced non-invasive differentiation between HCC and intrahepatic cholangiocarcinoma (ICC) (65).

Contrast-enhanced ultrasound (CEUS) has also benefited from AI-based approaches. A machine learning-based CAD system demonstrated enhanced diagnostic accuracy for FLL classification (66). Guo et al. employed deep canonical correlation analysis combined with multiple kernel learning in three-phase CEUS imaging, achieving a classification accuracy of 90.41% (67). Huang et al. developed a SVM-based CAD model capable of effectively distinguishing atypical HCC from focal nodular hyperplasia (68). Furthermore, incorporating ultrasound radiomics and clinical data in multi-phase CEUS imaging has further improved preoperative pathological grading of HCC, achieving superior AUC values compared to single-modality models (69).

### 4.2 Computed tomography-based methods

AI-based CT models have been developed to enhance lesion detection, classification, pathological grading, segmentation, and imaging protocol optimization. A hierarchical CNN framework achieved an average lesion detection accuracy of 82.8%, with a recall of 93.4% and an F1-score of 87.8%, demonstrating robust lesion identification. Additionally, its binary and six-class classification accuracies reached 82.5% and 73.4%, surpassing other neural networks and performing comparably to intermediate-level radiologists (70). In lesion classification, deep learning models analyzing dynamic contrast-enhanced CT images reported an AUC of 0.92 for distinguishing HCC from other malignant liver tumors (71). A CNN-based CAD system achieved a 98.3% classification accuracy and a tumor detection rate of 78%, underscoring AI's potential as a radiological diagnostic aid (72). Further advancements leveraging multiphase CT imaging include models integrating Hounsfield Unit density variations from four-phase CT scans with Faster R-CNN, R-FCN, and SSD networks, achieving a tumor detection accuracy of 100% and lesion classification accuracy of 95.1% (73). Additionally, Ponnoprat et al. combined CNN and SVM classifiers for distinguishing HCC from ICC, achieving an accuracy of 88% (74). Ensemble models integrating multiple classifiers have also been effective, with detection accuracies ranging from 98.39% to 100% and classification accuracies between 76.38% and 87.01%, surpassing individual classifier performances (75).

Beyond lesion classification, AI has been applied to non-invasive tumor grading, aiding in risk stratification. A study analyzing 13,920 quantitative imaging features extracted from three-phase CT scans developed a predictive model that achieved AUCs of 0.70 and 0.66 in discovery and validation cohorts, respectively, aiding in the identification of high-risk HCC patients (76). Another radiomics-based machine learning approach improved the model's AUC to 0.8014 when incorporating radiomic features, reinforcing AI's role in assessing tumor aggressiveness (77).

AI has also contributed significantly to automated tumor segmentation, a crucial step for treatment planning and response assessment. CNN-based segmentation models have outperformed conventional methods, with a Dice coefficient of 80.06%, precision of 82.67%, and recall of 84.34% (78). Another study proposed a multi-channel fully convolutional network that integrated multi-phase contrast-enhanced CT images, achieving a volume overlap error of 15.6 ± 4.3% on the 3Dircadb dataset, which further decreased to 8.1 ± 4.5% on the JDRD dataset, demonstrating enhanced accuracy and robustness (79). To further optimize segmentation, approaches incorporating voxel- and object-level models have significantly reduced false positives by 85%, achieving Dice coefficients of up to 69%, comparable to manual segmentation (80). Furthermore, adversarial training strategies have been implemented to refine segmentation models, yielding Dice coefficients of 68.4% while improving multiple segmentation metrics, including ASD, MSD, VOE, and RVD (81). The Successive Encoder-Decoder framework has further refined segmentation workflows, demonstrating Dice coefficients of 92% for liver segmentation and 75% for tumor prediction, emphasizing its clinical applicability (82).

AI has also been leveraged for CT imaging protocol optimization and recurrence monitoring, ensuring effective diagnosis while minimizing radiation exposure. Shi et al. compared three-phase and four-phase DCE-CT protocols for differentiating HCC from other FLLs, and they found that excluding the non-contrast phase did not significantly impact diagnostic performance, while substantially reducing radiation exposure, highlighting AI's role in balancing diagnostic accuracy with safety considerations (83). AI-assisted recurrence monitoring has also demonstrated significant improvements, with a CNN-based classifier integrating baseline and follow-up CT scans increasing new tumor detection rates from 72% to 86%, emphasizing AI's potential in long-term HCC surveillance (84).

### 4.3 Magnetic resonance imaging-based methods

AI applications in MRI have focused on lesion classification, automated detection, pathological grading, and segmentation. In HCC classification, deep learning models leveraging multi-sequence MRI have demonstrated high diagnostic accuracy. A study integrating dynamic contrast-enhanced MRI and T2-weighted images with clinical risk factors developed an automated system that achieved an overall classification accuracy of 0.77 for five common liver lesions (85). CNN-based models trained on large MRI datasets have outperformed radiologists, achieving an accuracy of 92% in lesion classification (86). Further improvements were seen when AI models incorporated additional MRI features, Oyama et al. employed texture analysis and topological data analysis on T1-weighted MRI, achieving an accuracy of 92% in distinguishing HCC from metastatic lesions (87). Trivizakis et al. leveraged 3D CNN to analyze diffusion weighted MRI, achieving an accuracy of 83% and outperforming conventional 2D CNN models, reinforcing the benefits of 3D volumetric feature extraction in liver lesion classification (88).

In automated HCC detection, a fine-tuned CNN model applied to hepatobiliary phase MRI achieved a sensitivity of 87% and specificity of 93%, underscoring its potential in early tumor detection (89). Zhen et al. developed multiple CNN-based models incorporating contrast-enhanced MRI, non-contrast MRI, and clinical data. Even when using non-contrast MRI alone, the model achieved an AUC of 0.946, which further improved to 0.985 when combined with clinical data, yielding a diagnostic concordance of 91.9%, demonstrating AI's ability to optimize non-invasive HCC assessment (90).

AI has also contributed significantly to lesion grading and segmentation, providing a means for automated Liver Imaging Reporting and Data System (LI-RADS) classification and tumor delineation. An AlexNet CNN model applied to multi-phase contrast-enhanced MRI for classifying LI-RADS grading achieved an accuracy of 90% and an AUC of 0.95, demonstrating performance comparable to expert radiologists (91). Liang et al. developed MRI-based radiomics models using random forest to differentiate hepatic epithelioid angiomyolipoma, HCC, and focal nodular hyperplasia, with an AUC of 0.999 for radiomics alone and 0.971 for an integrated model incorporating clinical features, surpassing conventional diagnostic methods (92). In addition, Bousabarah et al. introduced a CNN-based model trained on multiphasic contrast-enhanced MRI, demonstrating automated HCC detection and delineation (93).

Furthermore, advances in 3D CNN architectures have further improved the classification of atypical HCC. A study using multi-sequence MRI to differentiate typical and atypical HCC from non-HCC lesions reported an overall accuracy of 87.3%, with HCC classification sensitivity/specificity of 92.7%/82.0%, and non-HCC classification sensitivity/specificity of 82.0%/92.7%, achieving an AUC of 0.912 (94).

## 5 Conclusions

**Figure 2:**
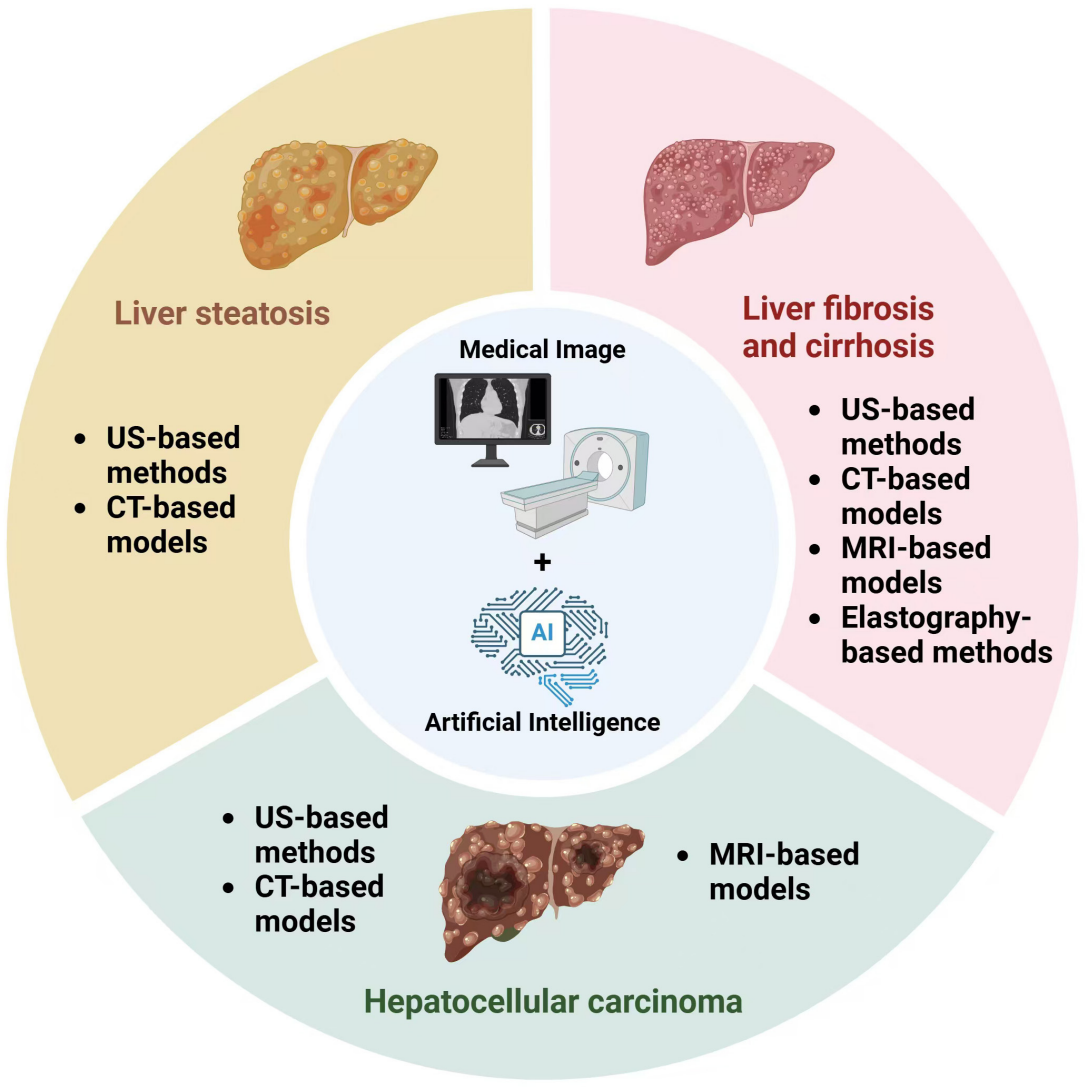
Application of AI image models in the diagnosis of fibrosis assessment, steatosis quantification, and hepatocellular carcinoma detection.

AI-driven imaging has significantly improved the detection, classification, and quantification of liver diseases, including fibrosis, steatosis, and HCC (Figure 2). By enhancing the diagnostic accuracy of US, CT, MRI, and elastography, AI has reduced observer variability and the need for invasive biopsies, offering a transformative approach to liver disease assessment.

However, challenges remain in standardizing imaging protocols, ensuring the generalizability of AI models across diverse populations and healthcare settings, and improving interpretability. Many AI models rely on institution-specific datasets, limiting their broader applicability. In the future, anonymized datasets across ethnicities, scanners, and disease etiologies should be established and used to validate these AI models. Researchers can also develop adaptive AI frameworks which can dynamically adjust to local imaging protocols or population characteristics. Besides, we can align AI validation with FDA/CE guidelines for medical devices. Additionally, the “black-box” nature of deep learning algorithms hinders clinical trust, necessitating the development of more explainable AI frameworks and rigorous validation through multicenter trials.

Although AI models can assist in the diagnosis of liver diseases to some extent, they cannot fully replace liver biopsy, particularly for fibrosis and steatosis, as liver biopsy remains the gold standard for diagnosing liver fibrosis and steatosis. While AI models for fibrosis staging and steatosis quantification have achieved sensitivities >85% and specificities >90%, they still fall short of biopsy in early-stage disease (F0-F1 fibrosis or <5% steatosis). In such cases, biopsy remains indispensable.

Besides, practical challenges such as computational resource requirements, radiologist acceptance, and regulatory approvals must also be addressed to facilitate integration into clinical workflows. For example, AI tools may require specialized hardware for real-time analysis, and their adoption depends on demonstrating cost-effectiveness and alignment with existing diagnostic pathways. Collaborative efforts between clinicians, data scientists, and policymakers are essential to overcome these barriers.

Despite these limitations, AI holds great promise for revolutionizing liver disease diagnostics. Emerging techniques, such as multi-modal AI integration, are poised to further advance the field. The combination of imaging data with clinical, genomic, and laboratory biomarkers will enhance diagnostic accuracy. For instance, we can couple radiomics features from MRI/CT with serum biomarkers or genetic risk scores, which may improve stratification of high-risk patients. Such multi-modal approaches will address the limitations of single-modality AI by capturing complementary biological insights. Additionally, integrating AI with real-time electronic health records (EHR) may enable dynamic risk prediction models. Future efforts should focus on enhancing model robustness, integrating AI with EHR and multi-omics data, and fostering interdisciplinary collaboration between clinicians, data scientists, and policymakers. With continued innovation, AI can bridge critical gaps in liver disease management, ultimately improving patient outcomes on a global scale.
